# First Broad-Range Serological Survey of Crimean–Congo Hemorrhagic Fever among Hungarian Livestock

**DOI:** 10.3390/v16060875

**Published:** 2024-05-29

**Authors:** Nóra Deézsi-Magyar, Béla Dénes, Bereniké Novák, Gyula Zsidei, Dániel Déri, Judit Henczkó, Bernadett Pályi, Zoltán Kis

**Affiliations:** 1 National Biosafety Laboratory, National Center for Public Health and Pharmacy, 1097 Budapest, Hungarynovak.berenike@nngyk.gov.hu (B.N.); deri.daniel@nngyk.gov.hu (D.D.);; 2 School of PhD Studies, Semmelweis University, 1091 Budapest, Hungary; 3 Department of Microbiology and Infectious Diseases, University of Veterinary Medicine Budapest, 1143 Budapest, Hungary; denesbela@yahoo.com; 4 National Laboratory of Infectious Animal Diseases, Antimicrobial Resistance, Veterinary Public Health and Food Chain Safety, University of Veterinary Medicine Budapest, 1078 Budapest, Hungary; 5 Veterinary Diagnostic Directorate, National Food Chain Safety Office, 1024 Budapest, Hungary; 6 European Research Infrastructure on Highly Pathogenic Agents (ERINHA-AISBL), B-1050 Brussels, Belgium; 7 Institute of Medical Microbiology, Faculty of Medicine, Semmelweis University, 1085 Budapest, Hungary

**Keywords:** Crimean–Congo hemorrhagic fever, tick-borne disease, serosurvey, livestock, Hungary

## Abstract

(1) Background: Crimean–Congo hemorrhagic fever (CCHF) is an emerging tick-borne disease endemic in Africa, Asia, the Middle East, and the Balkan and Mediterranean regions of Europe. Although no human CCHF cases have been reported, based on vector presence, serological evidence among small vertebrates, and the general human population, Hungary lies within high evidence consensus for potential CCHF introduction and future human infection. Thus, the aim of our pilot serosurvey was to assess CCHF seropositivity among cattle and sheep as indicator animals for virus circulation in the country. (2) Methods: In total, 1905 serum samples taken from free-range cattle and sheep in 2017 were tested for the presence of anti-CCHF virus IgG antibodies using commercial ELISA and commercial and in-house immunofluorescent assays. (3) Results: We found a total of eleven reactive samples (0.58%) from five administrative districts of Hungary comprising 8 cattle and 3 sheep. The most affected regions were the south–central and northwestern parts of the country. (4) Conclusions: Based on these results, more extended surveillance is advised, especially in the affected areas, and there should be greater awareness among clinicians and other high-risk populations of the emerging threat of CCHF in Hungary and Central Europe.

## 1. Introduction

Crimean–Congo hemorrhagic fever (CCHF) is one of the most widespread tick-borne diseases, potentially causing symptoms such as headache, fever, vomiting, back pain, joint pain, stomach pain, petechiae, and severe hemorrhagic manifestations in humans [1]. The reported fatality rate among hospitalized patients is 9–40%, and the number of annual cases is increasing worldwide [1,2]. The causative agent, the Crimean–Congo hemorrhagic fever orthonairovirus (CCHFV; recently renamed *Orthonairovirus haemorrhagiae*), is endemic to eastern and southern Europe, the Balkan region, the Middle East, Africa, and some parts of Asia [3,4,5]. CCHFV is an enveloped, negative-sense RNA virus with a tri-segmented viral genome [6]. In correspondence with the geographical distribution of the virus, which coincides with the expanding spatial spread of the principal tick vectors, CCHFV is a genetically diverse pathogen [1,3,5]. Three out of seven distinct lineages are circulating in Europe: the Europe-1 lineage (the most affected countries are Albania, Bulgaria, and Turkey), the Europe-2 lineage (reported in Greece and Turkey), and the Africa-3 lineage (recently affecting the western Mediterranean region) [3,5,6]. Studies have identified over 30 tick species as potential virus carriers (*Hyalomma* spp., *Rhipicephalus* spp., *Ornithodoros* spp., *Dermacentor* spp., *Ixodes* spp. ticks, etc.) [7,8]. Although *H. marginatum*, *H. lusitanicum*, and *H. rufipes* are proposed as the principal vectors, the spread of the Europe-2 lineage (as well as the Aigai virus (*Orthonairovirus parahemorrhagiae*) associated with less severe infections) is attributed to the *Rhipicephalus bursa* tick [7,8,9,10]. Due to global warming and the movement of migratory birds, the distribution of Mediterranean tick species has shifted further north in the previous decade [11,12]. *H. marginatum* and *H. rufipes* were identified at multiple times and places in Hungary between 2011 and 2021, and other potential tick vectors are also present in the country [13,14,15,16,17,18].

The natural cycle of CCHFV includes transovarial and transstadial transmission among ixodid ticks and involves different wild and domestic animals in Europe, such as hares, rodents, deer, cattle, sheep, and goats. The role of animals as virus reservoirs and sources of infection has been highlighted by many publications reporting the presence of asymptomatic viremia lasting up to 7–15 days [19,20]. As CCHFV is maintained through a tick–vertebrate–tick transmission cycle, the primary route for human infection is through tick bite or direct exposure to the infectious body fluids of humans or animals, including husbandry and removing infected ticks from animals [5,21,22,23,24]. As part of a successful One Health approach, sufficient information and a better understanding of the animal reservoirs and amplifying hosts and transmission routes of CCHFV are crucial to understanding human disease and outbreak potential [25]. At the population level, the presence of tick vectors and serological evidence among animals can indicate virus circulation in a specific area [21,22,23,24,26]. High-risk occupations for CCHFV infection include veterinarians, farmers, foresters, hunters, and abattoir workers who are in close proximity to livestock or can acquire tick bites [25]. In Hungary, CCHFV seropositivity has been detected in the last decade, both among animals (rodents and brown hares) and in the general human population [27,28,29]. CCHFV seroprevalence among animals is also increasing in neighboring countries, such as Romania and the Balkan states south of Hungary, where CCHFV is an endemic pathogen [21,30,31,32].

To obtain a more accurate picture of the potential distribution of CCHFV in the country, we aimed to perform the first broad-range serological survey focusing on large vertebrates such as cattle and sheep in Hungary.

## 2. Materials and Methods

### 2.1. Sample Selection

Blood specimens were taken from healthy free-range animals (cattle and sheep) as part of monitoring programs for various animal diseases in 2017. The samples were tested by the Laboratory of Immunology of the Veterinary Diagnostic Directorate, National Food Chain Safety Office (Budapest, Hungary). Subsequently, archived specimens were made available to the National Biosafety Laboratory for testing (National Center for Public Health and Pharmacy, Budapest, Hungary) at the National Food Chain Safety Office. Samples were received from 13 out of 20 statistical regions (NUTS3 regions; Nomenclature of Territorial Units for Statistics) of Hungary. In total, 1905 serum specimens were tested for the presence of anti-CCHFV IgG antibodies, comprising 1391 serum samples obtained from cattle and 514 samples from sheep.

### 2.2. Serological Testing

Serum specimens were stored at −20 °C before serological screening. After thawing, samples were inactivated at 56 °C in a water bath for 30 min and centrifuged at 7000× *g*. The sera of animals from the same district were combined into pools of a maximum of 5 samples.

To screen the cattle, the Cow Crimean–Congo Hemorrhagic Fever Virus IgG (CCHF-IgG) ELISA Kit (Abbexa Ltd., Cambridge, UK, catalog number abx050296) for detecting anti-CCHFV GP IgG antibodies was used according to the manufacturer’s instructions in a five-fold dilution. Optical density (OD) was measured using the absorbance reader (Tecan Trading AG, Mannedorf, Switzerland) at 450 nm. Samples were considered reactive if OD_sample_ ≥ cut off (calculated according to the manufacturer’s instruction as OD_negative control_ + 0.15). Equivocal pools were determined as near cut-off OD or a maximum of 10% below the cut-off OD value. Samples of the positive or equivocal pools were tested further individually with the same ELISA kit (in two-fold dilution) and two types of immunofluorescent assays. In-house indirect immunofluorescent slides (IIFA) containing the whole virus (CCHFV Afg09-2990 strain, Genbank acc. No. HM452305.1, HM452306.1, HM452307.1) were produced in Vero E6 cells and validated at the National Biosafety Laboratory, as previously described [29]. Individual serum samples were diluted in 1:20 in phosphate-buffered saline (PBS), added to IIFA slides, and incubated for 1 h at 37 °C. After incubation, slides were washed three times in PBS+Tween 20 (PBST) and stained with the FITC-conjugated Anti-Bovine IgG (whole molecule) secondary antibody (Merck KGaA, Darmstadt, Germany, catalog number B8395) diluted at 1:300. Slides were incubated for 30 min at 37 °C. For the positive control, anti-CCHFV IgG-positive polyclonal mouse serum was used in 1:160 dilution. For the EUROIMMUN CCHFV Mosaic 2 IgG immunofluorescence assay (EUROIMMUN Medizinische Labordiagnostika AG, Lübeck, Germany, catalog number FI 279a-2010-2 G), samples were diluted 1:20. The slides were stained with the FITC-conjugated Anti-Bovine IgG (whole molecule) secondary antibody at the dilution 1:300. Otherwise, the EUROIMMUN assay was performed according to the manufacturer’s instructions to detect reactivity.

To investigate the sheep serum pools, samples were pooled and diluted 1:20 in PBS for the in-house IIFA screening. Reactive samples were then tested further individually at the dilution 1:20 with two immunofluorescent assays. The in-house and the EUROIMMUN CCHFV Mosaic 2 IgG IIF assays were performed as described above, and slides were stained with the FITC-conjugated Anti-Sheep IgG (whole molecule) secondary antibody (Merck KGaA, Darmstadt, Germany, catalog number F7634) at the dilution 1:400.

Fluorescence read-out was performed with the Leica DMi8 (Leica Microsystems, Wetzlar, Germany) microscope system. Images were processed with the LAS X Life Science Microscope Software (Leica Microsystems, Wetzlar, Germany) software. Fluorescence was evaluated for anti-CCHFV IgG-specific staining compared to the positive control by two independent individuals.

End-point serum titration of the individual positive samples was performed in a two-fold dilution (dilution range 1:10–1:640) using the in-house IIFA. Among the cattle samples, those that showed reactive results in ELISA and both IIFA assays were considered positive, and among the sheep samples, positivity was concluded if reactivity was detected in both in-house and commercial IIFAs.

### 2.3. Data Analysis

Prevalence was defined as the percentage of anti-CCHFV IgG-positive samples in the study groups with 95% confidence intervals (CIs) in each NUTS3 region and district using GraphPad Prism 9.5.0 software (GraphPad Software Inc., Boston, MA, USA). To describe the geographical distribution of seropositive animals at the district level, results were visualized using Quantum GIS 3.0 software with district administrative borders obtained via the OpenStreetMap database [33].

## 3. Results

### 3.1. Sample Pooling and Screening

In total, 1905 serum samples were tested for anti-CCHFV IgG antibodies. From the cattle serum specimens (*n* = 1391), 287 pools were drawn, including 2–5 samples per pool based on geolocation. From the sheep serum specimens, 105 pools with 2–5 samples per pool were created. During the first screening, six pools of cattle samples and one pool of sheep samples were reactive (*n* = 3) or equivocal (*n* = 4). Serum specimens of the reactive or equivocal pools were tested further individually. Among the cattle, a total of eight samples (from five pools) showed anti-CCHFV IgG reactivity in all three assays (the Abbexa ELISA Kit, the in-house IIFA, and the EUROIMMUN Mosaic IIFA) with titers of 1:20 and 1:40 (Table 1). Among the sheep, three reactive samples (with both IIFA assays) originated from a single sample pool with final anti-CCHFV IgG titers of 1:160 and 1:320 (Table 2). In the seventh pool, which gave equivocal results in the first screening, no individual sample was confirmed to be reactive (Supplementary Materials: Table S1 and Figures S1–S3).

### 3.2. Geographical Distribution

**Figure 1:**
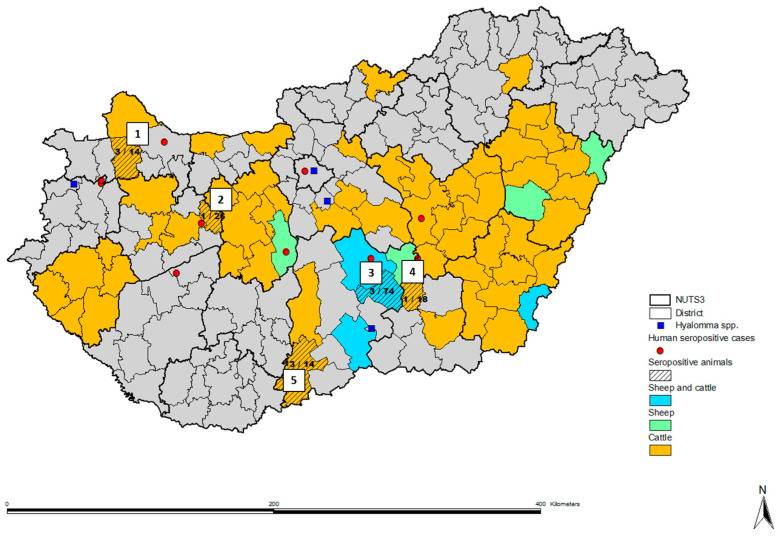
Map of Hungary showing administrative districts. Cattle and sheep serum samples were received from districts highlighted in orange and green, respectively. From four districts, both cattle and sheep samples were available for testing (highlighted in blue). The whole NUTS3 region is highlighted if the origin of the samples was known only at the NUTS3 level. Streaked districts represent regions with seropositive animals (1—Csorna, Győr–Moson–Sopron county; 2—Várpalota, Veszprém county; 3—Kiskunfélegyháza, Bács–Kiskun county; 4—Csongrád, Csongrád county; 5—Baja, Bács–Kiskun county). Red dots represent data from a previous study with identified CCHFV seropositive human blood donors [29]. Blue squares show historical data on the identification of Hyalomma spp. ticks in Hungary [13,15,16,17,18].

Samples were received from 13 NUTS3 regions, including 34 districts, mainly from the eastern and central parts of Hungary, with only a limited number of samples being available from the western and northern regions (Figure 1). For 514 cattle samples, the origin was known only at the NUTS3 region level. Among these cattle, seropositive samples were identified in five districts from four NUTS3 regions, one animal from the Csongrád district (Csongrád county), three animals from the Baja district (Bács–Kiskun county), three animals from Csorna district (Győr–Moson–Sopron county), and one cattle from Várpalota district (Veszprém county), showed seropositivity (Figure 1).

Sheep serum samples were received from four NUTS3 regions, including eight districts. We found three seropositive samples from the Kiskunfélegyháza district (Bács–Kiskun county) (Figure 1).

The most affected NUTS3 region was Bács–Kiskun in the south–central part of Hungary, with an average seropositivity of 1.8% (95% CI 1.60–1.99%) among the tested animal population. The northwestern part of the country was also affected; samples obtained from cattle in Győr–Moson–Sopron county showed reactivity, and we also found seropositive cattle in Várpalota district (Veszprém county). The overall seropositivity of the tested animals in the affected NUTS3 regions was 2.33% (95% CI 2.12–2.54%).

## 4. Discussion

Given the emergence of CCHFV in the European region, which is now affecting new geographical areas and causing autochthonous human infections [34], our retrospective study aims to gain information and assess CCHFV seropositivity among vertebrates in Hungary. Sera taken in 2017 from healthy free-range cattle and sheep were tested for anti-CCHFV IgG.

According to the latest technical report published by the European Centre for Disease Prevention and Control in December 2023, and based on vector presence and serological evidence among small vertebrates and the general human population, there is a high evidence consensus for potential CCHFV introduction and future human infection in Hungary [13,14,15,16,17,18,27,28,29,35]. Furthermore, in terms of the One Health approach, Hungary is now classified as a level 3 country with no CCHF cases reported and no robust surveillance established; however, available data point toward the possibility of undetected CCHF cases and/or potential virus introduction [36,37,38].

Serological surveys serve as the predominant source of information to monitor areas with natural virus transmission [24,35]; a local study focusing on the southeastern region of the country found serological evidence of CCHFV among European brown hares (Lepus europaeus) collected between 2008 and 2009 [27]. Another survey testing wild rodents captured between 2011 and 2013 in the Mecsek Mountain region found 0.96% seroprevalence using dot-blot and immunofluorescence assays [28]. Furthermore, according to our previous systematic screening in Hungary of 2700 healthy blood donors, we found 0.37% CCHFV seroprevalence in the tested human population [29].

The number of seropositive animals is also increasing in the neighboring countries of Hungary. In southern Romania, a study performed between 2019 and 2020 assessed the seroprevalence of CCHFV in 250 sheep and goats using ELISA; the results showed an overall 37.7% antibody positivity [30]. In Albania, a study published in 2014 reported 46% seropositivity in sheep and 28% in goats [21]. In North Macedonia, the seropositivity among cattle was estimated to be as high as 80% in the northeastern part of the country, including 75% in sheep and 59% in goats [31]. In Bulgaria, high CCHFV seroprevalence was also detected in cattle, goats, and sheep, with 19.6%, 22.7%, and 7.7% IgG positivity, respectively [32]. Gaining data on the levels of seropositivity among animals is particularly important from the One Health aspect as well, as several cases of primary human infections are described where the person came into direct contact with the infectious body secretions of the infected animal or removed an infected tick and consequently became infected with CCHFV [23,39,40,41]. Furthermore, secondary nosocomial infections have also been described where the index case occurred due to direct contact with an infected animal or tick bite. Then, the medical staff involved in the treatment also acquired CCHFV infection [42]. According to a systematic review published in 2019, the global trend of CCHFV seroprevalence in livestock showed an increase over time (between 1969 and 2018), with 10.0–27.0%, 19.0–41.0%, and 0.5–37.0% for camels, sheep, and livestock, respectively. In cattle and goats, it exhibited a slightly increasing trend, with 16.5–21.0 and 22.0–27.0%, respectively [43]. The tendency of CCHFV infections attributed to secretion exposure also increased between 2009 and 2017 in the CCHFV endemic areas and ranged between 18.5 and 34.0% [43]. Seropositivity in the general human population in endemic countries (Iran, Turkey, Greece, Bulgaria, Georgia, etc.) was estimated to be 2.5–5.5%, while among high-risk groups for infection (including foresters, hunters, abattoir workers, livestock handlers, butchers, or other occupations requiring the handling of domestic livestock or posing an enhanced risk of tick bites), the total mean seroprevalence was 16.5–36.5% [24,25,43]. Furthermore, investigations into human infections in endemic areas often revealed elevated CCHFV seroprevalence in local livestock [24,43].

In the present study, we found CCHFV seropositive animals in the south–central and northwestern regions of Hungary to have an overall seropositivity of 2.33% in the affected NUTS3 regions. Regarding geographical distribution, these data correlate with our previous study determining CCHF seroprevalence among the general human population and are also comparable with the historical reports of Hyalomma ticks in Hungary (Figure 1). The western and central parts of the country are considered tick-endemic areas, including those where we identified CCHFV seropositive animals [13,14,15,16,17,18,29].

According to the CDC, there is no standardized serological method for the human diagnosis of CCHF; however, immunofluorescent assays based on the whole virus are sensitive methods [1,42]. A study published in 2014 states that most European reference laboratories with the capacity to diagnose CCHFV infection use in-house IIFA kits for serology, and fifty percent of the laboratories that use commercially available ELISA kits also claim to combine the results of commercial kits with in-house serological assays, which may compensate for the potentially reduced sensitivity caused by the antigenic diversity of CCHFV [44]. In the present study, due to the high number of samples, sera from the same geolocations were tested in pools containing two to five samples per pool, which may lead to reduced sensitivity. To overcome this, we used multiple serological assays with low initial sample dilutions (1:5 for the ELISA kit and 1:20 for the IIFA kits) to increase sensitivity.

To the best of our knowledge, this is the first retrospective CCHFV serosurvey focusing on free-range vertebrates in Hungary covering a significant geographical area (13 of 20 administrative NUTS3 regions). Although the serum samples were taken in 2017, and the distribution of the tested samples is not representative of the entire country, we obtained essential data on the CCHFV serological status in Hungary from a few years ago. In relation to the increasing seropositivity in the surrounding countries of Hungary, and based on other risk factors, such as vector presence, climatic features, and serological evidence, the probability of the potential introduction and subsequent human CCHFV infection in the country is significant [13,14,15,16,17,18,27,28,29,35]. The results of this pilot serosurvey can serve as a basis for a systematic, cross-sectorial serosurveillance program focusing on at-risk human, animal, and tick populations. As the next step, we would like to assess the current seroprevalence in the country to gain important information regarding the spatio-temporal emergence of CCHFV since 2017. We also aim to perform active and comprehensive surveillance focusing on the principal tick vectors present and the CCHFV strains that potentially circulate in the affected geographical areas of Hungary. Moreover, our results highlight the importance of raising awareness among healthcare workers and other at-risk populations of the emerging threat of CCHFV in Hungary and Central Europe.

## Figures and Tables

**Table 1:** Results of the serological screening for anti-CCHFV IgG antibodies among cattle, including the number of tested samples and the number of reactive samples for each NUTS3 region and district of origin, and the final anti-CCHFV IgG titer obtained with the IIFA tests. Samples that gave reactive results in all three assays (Abbexa ELISA Kit, in-house IIFA, and EUROIMMUN Mosaic IIFA) were classified as seropositive.

NUTS3 Region	District of Origin	Number of Tested Animals	Number of Positive Animals	IIFA Titers
Bács–Kiskun	Kecskemét	14	0	n.a.
	Baja	14	3	1:40
	Kiskunfélegyháza	14	0	n.a.
	Kiskunhalas	28	0	n.a.
	Kalocsa	2	0	n.a.
Békés	Gyula	65	0	n.a.
	Mezőkovácsháza	18	0	n.a.
	Sarkad	1	0	n.a.
	unknown	50	0	n.a.
Borsod–Abaúj–Zemplén	Szerencs	5	0	n.a.
Csongrád	Hódmezővásárhely	28	0	n.a.
	Csongrád	18	1	1:40
Győr–Moson–Sopron	Csorna	14	3	1:20–1:40
	Mosonmagyaróvár	1	0	n.a.
Hajdú–Bihar	unknown	11	0	n.a.
Jász–Nagykun–Szolnok	Törökszentmiklós	54	0	n.a.
	Jászberény	13	0	n.a.
	Szolnok	79	0	n.a.
	Karcag	3	0	n.a.
	unknown	457	0	n.a.
Fejér	Mór	1	0	n.a.
	Sárbogárd	2	0	n.a.
	unknown	30	0	n.a.
Komárom–Esztergom	Komárom	30	0	n.a.
	Esztergom	12	0	n.a.
Nógrád	Salgótarján	15	0	n.a.
Veszprém	Pápa	6	0	n.a.
	Ajka	28	0	n.a.
	Veszprém	17	0	n.a.
	Várpalota	26	1	1:40
Pest	Aszód	14	0	n.a.
	Monor	57	0	n.a.
	Dabas	215	0	n.a.
	Cegléd	16	0	n.a.
Zala	unknown	33	0	n.a.
SUM		1391	8	

n.a.: not applicable.

**Table 2:** Results of the serological screening for anti-CCHFV IgG antibodies among sheep, including the number of tested samples and the number of reactive samples for each NUTS3 region and district of origin, and the final anti-CCHFV IgG titer obtained with the IIFA tests. Samples that gave reactive results in both assays (in-house IIFA and EUROIMMUN Mosaic IIFA) were classified as seropositive.

NUTS3 Region	District of Origin	Number of Tested Animals	Number of Positive Animals	IIFA Titers
Bács–Kiskun	Tiszakécske	44	0	n.a.
	Kiskunfélegyháza	60	3	1:160–1:320
	Kecskemét	96	0	n.a.
	Kiskunhalas	62	0	n.a.
Békés	Gyula	62	0	n.a.
Hajdú–Bihar	Nyíradony	95	0	n.a.
	Püspökladány	35	0	n.a.
Fejér	Dunaújváros	60	0	n.a.
SUM		514	3	

n.a.: not applicable.

## Data Availability

Data is contained within the article or Supplementary Materials.

## Supplementary Materials

The following supporting information can be downloaded at: https://www.mdpi.com/article/10.3390/v16060875/s1, Table S1. Detailed results of the reactive pools and individual samples; Figure S1. Results (OD values) obtained with Cow Crimean–Congo Hemorrhagic Fever Virus IgG (CCHF-IgG) ELISA kit; Figure S2. Results obtained with the different immunofluorescent assays; Figure S3. Detection of anti-CCHFV IgG with in-house (produced at the NCPHP NBL) IIFA slides.
