# Supplementary material for: First Broad-Range Serological Survey of Crimean–Congo Hemorrhagic Fever among Hungarian Livestock
Source: Viruses. 2024 May 29;16(6):875. doi: 10.3390/v16060875 (PMC11209279; doi:10.3390/v16060875)
Supplement: Supplementary file 1 [file viruses-16-00875-s001.zip › viruses-3002475-supplementary.pdf]

Supplementary materials

S. Table 1. Detailed results of the reactive pools and individual samples by the initial screening and verification testing with ELISA or in-house IIFA testing. * Cattle sample pools were screened with the Cow Crimean–Congo Hemorrhagic Fever Virus IgG (CCHF-IgG) ELISA Kit (Abbexa Ltd, Cambridge, United Kingdom). To test sheep samples pools, the in-house IIFA slides stained with FITC-conjugated Anti-Sheep IgG (whole molecule) were used. n.a.: not applicable

	NUTS3 of origin	District of origin	ELISA/IIFA of the pool*	Pool/sample number	ELISA result (titer)	in-house IIFA (titer)	Euroimmun IIFA	Final result
Cattle	Pest	Monor	Equivocal	C127/1	negative	<1:20 negative	negative	negative
				C127/2	negative	<1:20 negative	negative	negative
				C127/3	negative	<1:20 negative	negative	negative
				C127/4	negative	<1:20 negative	negative	negative
				C127/5	negative	<1:20 negative	negative	negative
	Bács-Kiskun	Baja	Reactive	C135/1	1:20 reactive	1:40 reactive	reactive	positive
				C135/2	1:40 reactive	1:40 reactive	reactive	positive
				C135/3	1:20 reactive	1:40 reactive	reactive	positive
				C135/4	negative	<1:20 negative	indeterminate	negative
				C135/5	negative	<1:20 negative	negative	negative
	Győr-Moson-Sopron	Csorna	Reactive	C36/1	negative	<1:20 negative	negative	negative
				C36/2	negative	<1:20 negative	negative	negative
				C36/3	1:20 reactive	1:40 reactive	reactive	positive
				C36/4	negative	<1:20 negative	negative	negative
				C36/5	1:40 reactive	1:20 reactive	reactive	positive
	Győr-Moson-Sopron	Csorna	Equivocal	C37/1	negative	<1:20 negative	negative	negative
				C37/2	negative	<1:20 negative	negative	negative
				C37/3	negative	<1:20 negative	negative	negative
				C37/4	1:5 reactive	1:20 reactive	reactive	positive
				C37/5	negative	<1:20 negative	negative	negative
	Veszprém	Várpalota	Equivocal	C57/1	1:20 reactive	1:40 reactive	reactive	positive
				C57/2	negative	<1:20 negative	negative	negative
	Csongrád	Csongrád	Equivocal	C83/1	negative	<1:20 negative	negative	negative
				C83/2	negative	<1:20 negative	negative	negative
				C83/3	1:10 reactive	1:40 reactive	reactive	positive
				C83/4	negative	<1:20 negative	negative	negative
				C83/5	negative	<1:20 negative	indeterminate	negative
Sheep	Bács-Kiskun	Kiskunfélegyháza	Reactive	S86/1	n.a.	1:160 reactive	reactive	positive
				S86/2	n.a.	<1:20 negative	negative	negative
				S86/3	n.a.	<1:20 negative	negative	negative
				S86/4	n.a.	1:160 reactive	reactive	positive
				S86/5	n.a.	1:320 reactive	reactive	positive

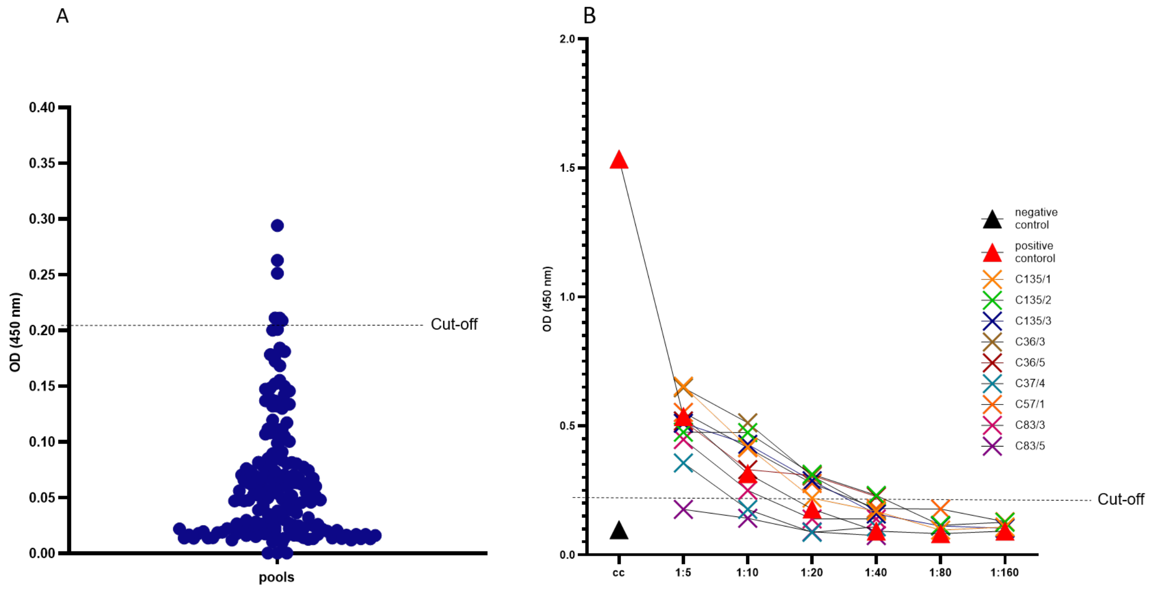

S. Figure 1. Results (OD values) obtained with Cow Crimean–Congo Hemorrhagic Fever Virus IgG (CCHF-IgG) ELISA kit for the **A.** pooled and **B.** individual samples of the reactive pools. ELISA testing was performed according to manufacturer's instructions with pool samples diluted in 5-fold. Reactive individual samples were two-fold titrated (dilution range 1:5 – 1:160) and end-point titer was determined compared to cut-off OD to validate results. Read-out was performed at 450 nm. Cut-off value was determined as $OD_{\text{negative control}}$ [marked as ▲ in the figure] + 0.15 according to the manufacturer's instructions. Test is valid if $OD_{\text{positive control}} \geq 1.0$. Sample pools and individual samples with OD ≥ cut-off value were considered reactive. OD values of the reactive samples (in dilution 1:5) were significantly higher compared to the OD of negative samples (unpaired t-test, $P < 0.0001$, GraphPad Prism 9.5.0 software).

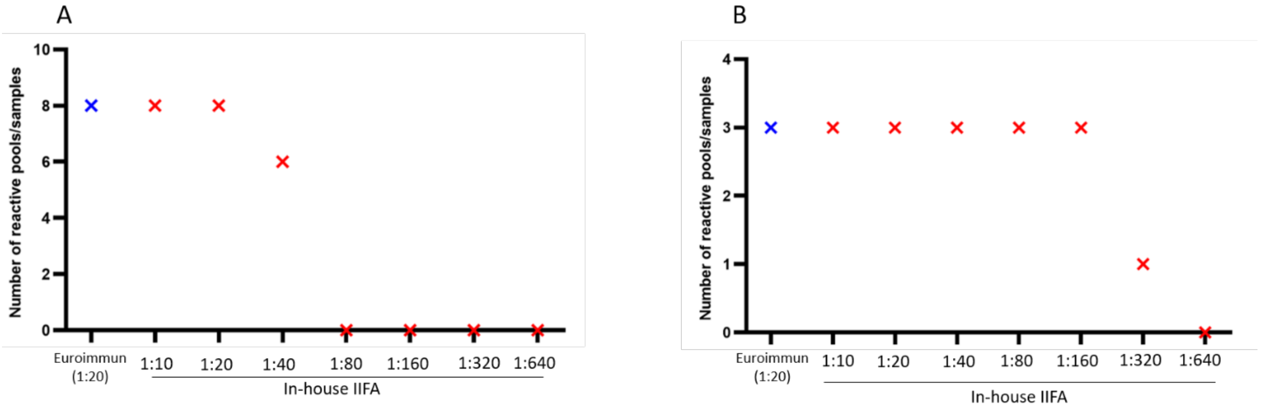

S. Figure 2. Results obtained with the different immunofluorescent assays among **A**. cattle, **B**. sheep serum samples. Individual samples were diluted 1:20 for the EUROIMMUN IIFA testing. To determine the final anti-CCHF IgG antibody titer of the individual samples, two-fold serial dilution was tested in the range of 1:10 – 1:640 (shown on *x*-axis) using the in-house IIFA. Number of reactive samples at each dilution rate is

shown in the graph (y-axis). Among the cattle samples, those that showed clear reactive results in all three tests were considered positive (n = 8), and among the sheep samples, positivity was confirmed if reactivity was detected in both in-house and commercial IIFAs (n = 3).

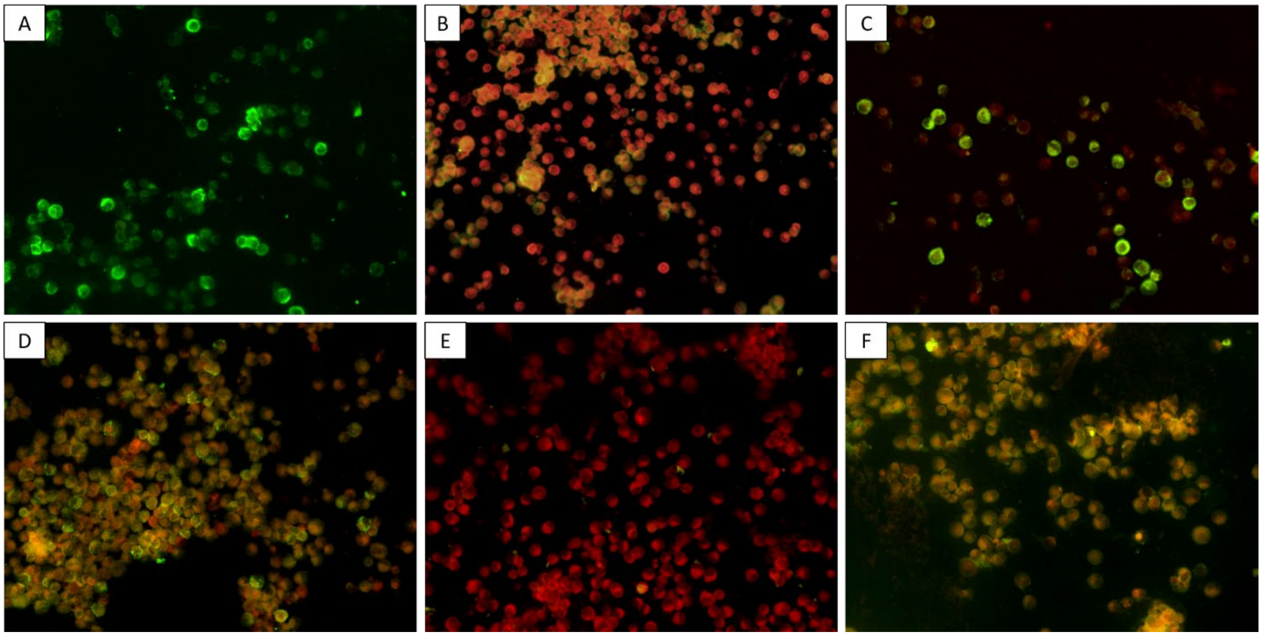

S. Figure 3. Detection of anti-CCHFV IgG with in-house (produced at the NCPHP NBL) IIFA slides containing whole-virus (CCHFV Afg09-2290 strain) infected Vero E6 cells. **A.** Reactive sheep sample pool (S86, dilution 1:20) with high background. Samples within the pool were tested further individually to verify anti-CCHFV IgG positivity. **B**. Negative sheep sample pool (dilution 1:20) with moderate background noise. **C**. Reactive sheep serum sample (S86/5, dilution 1:80). **D**. Positive cattle sample (C135/1, dilution 1:20). **E**. Negative cattle sample (dilution 1:20). **F**. Indeterminate cattle sample (C83/5, dilution 1:20) with high background, sample was discarded and concluded as seronegative (see S. Table 1). Immunofluorescence slides were stained with the respective secondary antibody (FITC-conjugated Anti-Sheep IgG (dilution 1:400) or FITC-conjugated Anti-Bovine IgG (dilution 1:300)). Fluorescence read-out was performed with the Leica DMi8 (Leica Microsystems, Wetzlar, Germany) microscope system. Images were processed with the LAS X Life Science Microscope Software (Leica Microsystems, Wetzlar, Germany) software. Fluorescence was evaluated for anti-CCHFV IgG-specific staining compared to the positive control (anti-CCHFV IgG positive polyclonal mouse serum) by two independent individuals. Photo: Leica DMi8 20x.
